# Impact of the Geriatric Nutritional Risk Index on In-Hospital Mortality and Length of Hospitalization in Patients with Acute Decompensated Heart Failure with Preserved or Reduced Ejection Fraction

**DOI:** 10.3390/jcm9041169

**Published:** 2020-04-19

**Authors:** Susumu Hirose, Sakiko Miyazaki, Shoichiro Yatsu, Akihiro Sato, Sayaki Ishiwata, Hiroki Matsumoto, Jun Shitara, Azusa Murata, Takao Kato, Shoko Suda, Yuya Matsue, Masaru Hiki, Atsutoshi Takagi, Hiroyuki Daida, Takatoshi Kasai

**Affiliations:** 1 Department of Cardiovascular Medicine, Juntendo University Graduate School of Medicine, 2-1-1, Hongo, Bunkyo-ku, Tokyo 113-8421, Japan; s-hirose@juntendo.ac.jp (S.H.); syatsu@juntendo.ac.jp (S.Y.); ak-sato@juntendo.ac.jp (A.S.); s-ishiwata@juntendo.ac.jp (S.I.); hmatsumo@juntendo.ac.jp (H.M.); jshitara@juntendo.ac.jp (J.S.); azmurata@juntendo.ac.jp (A.M.); tkatou@juntendo.ac.jp (T.K.); ssuda@juntendo.ac.jp (S.S.); yuya8950@gmail.com (Y.M.); ma-hiki@juntendo.ac.jp (M.H.); a.taka@juntendo.ac.jp (A.T.); daida@juntendo.ac.jp (H.D.); kasai-t@mx6.nisiq.net (T.K.); 2 Cardiovascular Respiratory Sleep Medicine, Juntendo University Graduate School of Medicine, Ochanomizu KS building #303, 3-3-1, Hongo, Bunkyo-ku, Tokyo 113-8421, Japan; 3 Cardiovascular Management and Remote Monitoring, Juntendo University Graduate School of Medicine, Ochanomizu KS building #303, 3-3-1, Hongo, Bunkyo-ku, Tokyo 113-8421, Japan

**Keywords:** acute decompensated heart failure, Geriatric Nutritional Risk Index, length of hospital stay, malnutrition

## Abstract

In patients with heart failure (HF), the impact of the Geriatric Nutritional Risk Index (GNRI) on in-hospital mortality and length of hospital stay remains unclear. We aimed to identify the factors associated with increased in-hospital mortality and longer length of hospital stay considering the GNRI in acute decompensated HF with reduced and preserved ejection fraction (HFrEF and HFpEF, respectively). Patients with acute decompensated HF who were admitted to our institution between 2007 and 2011 were investigated. A total of 451 (201, HFrEF; 250, HFpEF) patients were divided into the following: patients with GNRI < 92 and ≥92. In HFrEF, there were no significant differences in in-hospital mortality and length of hospital stay between patients with GNRI < 92 and ≥92 (median (interquartile range), 24.0 (23.8) days and 20.0 (15.0) days, respectively, *p* = 0.32). In HFpEF, despite no differences in in-hospital mortality, patients with GNRI < 92 had significantly longer length of hospital stay than those with GNRI ≥ 92 (median (interquartile range), 20.0 (22.3) days and 17.0 (16.0) days, respectively, *p* = 0.04). In HFpEF, GNRI < 92, along with lower hemoglobin, higher B-type natriuretic peptide, and elevated C-reactive protein levels, were the independent factors for longer length of hospital stay. Among patients with acute decompensated HF, assessment of nutritional status with GNRI is useful for stratifying patients at high risk for longer length of hospital stay in HFpEF but not in HFrEF. These observations are particularly important when considering the increasing elderly population and prevalence of HFpEF.

## 1. Introduction

Despite recent advancements in the treatments for heart failure (HF), HF remains one of the major causes of increased risks of hospitalization and mortality [1]. In Japan, the prevalence of HF is increasing [2] in association with an increasing elderly population [3]. Multiple comorbidities are a well-known condition in the elderly population [4] and are generally associated with a greater risk of death and longer length of hospital stay. Certainly, in Japan, hospitalized patients with acute decompensated HF have multiple comorbidities [5,6,7]; an in-hospital mortality rate of approximately 5%, which remains substantial; and a median length of hospital stay of 14–21 days [5], which is substantially longer than that in any Western and Asian countries in association with a different socialized medical system in Japan [8]. HF with preserved ejection fraction (EF) (HFpEF) is prevalent in elderly HF patients and remains a major concern considering its limited treatment options [9]. Thus, it is important to identify the comorbid conditions or factors on admission that are associated with an increased risk of in-hospital mortality and longer length of hospital stay in patients hospitalized due to acute decompensated HF, specifically patients with HFpEF.

Malnutrition is frequently observed and an important risk factor of poor outcomes in patients with HF [10]. Although no nutritional evaluation method for patients with HF has yet been established, it has been reported that the Geriatric Nutritional Risk Index (GNRI) is useful for predicting the mortality risk of elderly HF patients [11]. The GNRI is a simple and objective nutritional index that uses the ideal body weight ratio and serum albumin, and GNRI < 92 is generally used for evaluating the risk of morbidity and mortality in hospitalized elderly patients [12]. However, the impact of the GNRI on short-term outcomes such as in-hospital mortality and LOHS remain uncertain. In particular, the effects of GNRI on such clinical outcomes in patients with HFpEF, which is assessed separately with HF with reduced EF (HFrEF), are of significant interest.

This study aimed to identify the factors associated with increased in-hospital mortality and longer length of hospital stay by considering the GNRI on admission in acute decompensated HF patients with HFrEF and HFpEF, respectively.

## 2. Materials and Methods

### 2.1. Subjects

This was a single-center, retrospective, observational study using a prospective database. The Institutional Review Board of Juntendo University Hospital approved the study protocol, and informed consent was obtained from all patients. Patients who were admitted to the cardiac intensive care unit in Juntendo University Hospital for acute decompensated HF from January 2007 to December 2011 were included in the study. Acute decompensated HF was defined based on the modified Framingham criteria [13]. Patients with acute coronary syndrome, active malignancy, and hemodialysis and patients undergoing surgery during hospitalization were excluded from this study. Additionally, patients who had missing data at baseline were excluded.

### 2.2. Data Collection

Baseline data were prospectively collected at the time of initial hospital admission. The medical history was obtained from a review of the patients’ clinical charts. Standard two-dimensional echocardiography was performed for each patient. Left ventricular EF (LVEF) was calculated according to the modified Simpson method. In the present study, HFpEF and HFrEF were defined as LVEF ≥ 40% and <40%, respectively [14]. Estimated glomerular filtration rate (eGFR) was calculated using the Modification of Diet in Renal Disease equation with a Japanese coefficient from baseline serum creatinine levels [15]. GNRI was calculated from the serum albumin and body mass index obtained on hospital admission as follows: 14.89 × serum albumin (g/dL) + 41.7 × body mass index/22 [12]. Low GNRI (<92) was defined as moderate-to-severe nutritional risk, and high GNRI (≥92) was defined as low or no nutritional risk, according to a previous report [12]. The incidence of in-hospital mortality and the date of discharge were prospectively collected. The endpoints of interest were in-hospital mortality and length of hospital stay. To assess the effects of the factors associated with length of hospital stay separately from in-hospital mortality, patients with in-hospital mortality were excluded from the analysis regarding length of hospital stay.

### 2.3. Statistical Analyses

Normally and non-normally distributed continuous variables are expressed as mean ± standard deviation and median and interquartile range (IQR), respectively. Continuous variables were compared using the unpaired Student’s t-test or Mann–Whitney U-test when appropriate. Categorical variables are expressed as numbers and percentages, and the chi-squared test or Fisher’s exact test was used for comparisons. Factors associated with in-hospital mortality and length of hospital stay were determined by logistic regression analysis in patients with HFrEF and HFpEF, respectively. In these analyses, C-reactive protein (CRP) levels were treated as categorical variables (i.e., > or ≤1.0 mg/dL) to separate the effects of elevated CRP levels (i.e., >1.0 mg/dL) from subjects with subclinical/healthy CRP levels (≤1.0 mg/dL) [16]. Plasma B-type natriuretic peptide (BNP) was treated as a log-transformed continuous variable because the values were skewed. Furthermore, because of the skewed distribution, length of hospital stay was also used as a categorical variable (i.e., > or ≤ geometric mean of length of hospital stay). Univariate logistic regression analysis was performed by considering the following independent variables: age, sex, history of prior HF, ischemic etiology, atrial fibrillation, diabetes mellitus, hypertension, current smokers, New York Heart Association (NYHA) class ≥ III, serum sodium level, serum potassium level, hemoglobin level, eGFR, elevated CRP level, plasma BNP level, use of medications on admission, and GNRI < 92, in addition to in-hospital mortality or longer hospital stay as dependent variables. Independent variables with *p* values < 0.15 in the univariate analysis were included in the multivariate analysis. A two-sided *p*-value < 0.05 was considered statistically significant. All analyses were performed using the EZR version 1.37 (Saitama Medical Center, Jichi Medical University, Saitama, Japan), which is a graphical user interface for R (R Foundation for Statistical Computing, Vienna, Austria).

## 3. Results

### 3.1. Study Population

**Figure 1:**
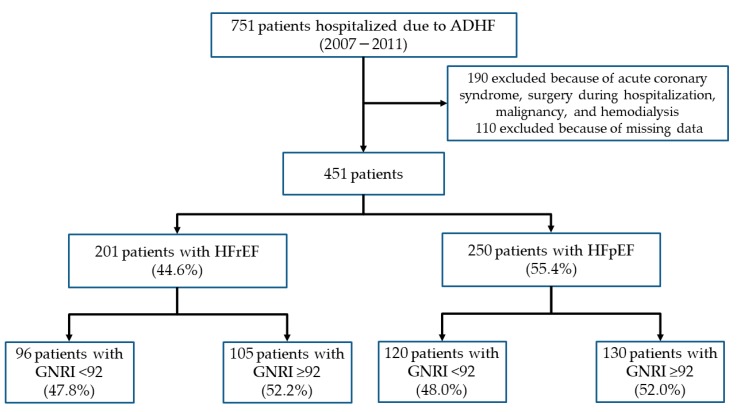
Flow diagram of this study. A total of 751 patients were admitted to the cardiac intensive care unit due to acute decompensated heart failure. Among these, 190 patients who had acute coronary syndrome and/or had undergone cardiac surgery, who had malignancy, and who were on hemodialysis were initially excluded, as were 110 patients who had missing data. The 451 eligible patients were subsequently divided into the HFrEF and HFpEF groups. Abbreviations: HFpEF, heart failure with preserved ejection fraction; HFrEF, heart failure with reduced ejection fraction.

A total of 751 patients were admitted to the cardiac intensive care unit due to acute decompensated HF. Patients with acute coronary syndrome and active malignancy, who underwent surgery during hospitalization, and who were on long-term hemodialysis were excluded from the study (*n* = 190). Additionally, 110 patients whose data for GNRI and/or others were not available at the time of initial hospital admission were excluded from the analysis. Thus, 451 patients, including 201 with HFrEF and 250 with HFpEF, were included in the study (Figure 1). Patients with HFpEF were significantly older than those with HFrEF (71.5 ± 13.1 years versus 67.1 ± 14.3 years, respectively, *p* < 0.001). However, there were no significant differences in the other variables, including GNRI, between the two groups (GNRI, 93.1 ± 13.6 in HFrEF and 92.3 ± 12.5 in HFpEF, *p* = 0.51). Overall, in-hospital mortalities were observed in 41 (14 (7.0%) in HFrEF and 27 (10.8%) in HFpEF) patients (9.1%) (*p* = 0.18). The length of hospital stay in patients without in-hospital mortality was significantly greater in the HFrEF group than in the HFpEF group (median (IQR), 21.0 (18.0) days versus 18.0 (18.5) days, respectively, *p* = 0.048).

### 3.2. Heart Failure with Reduced Ejection Fraction

#### 3.2.1. Baseline Characteristics

Characteristics of patients with HFrEF (*n* = 201) are shown in Table 1. Among them, 96 and 105 patients showed GNRI < 92 (47.8%) and GNRI ≥ 92 (52.2%), respectively. Patients with GNRI < 92 were significantly older and had lower hemoglobin levels, higher BNP levels, and lower body mass index, serum albumin levels and GNRI than those with GNRI ≥ 92 (Table 1).

#### 3.2.2. In-Hospital Mortality

Of the 14 in-hospital mortalities, 10 (10.4%) were observed in patients with GNRI < 92 and 4 (3.8%) in patients with GNRI ≥ 92 (*p* = 0.10). According to the univariate logistic regression analyses, GNRI < 92, use of diuretics, and low serum sodium and hemoglobin levels showed *p* values < 0.15. According to the multivariate analysis, only serum sodium and hemoglobin levels were determined as independent factors associated with increased in-hospital mortality (Table 2).

#### 3.2.3. Length of Hospital Stay

**Figure 2:**
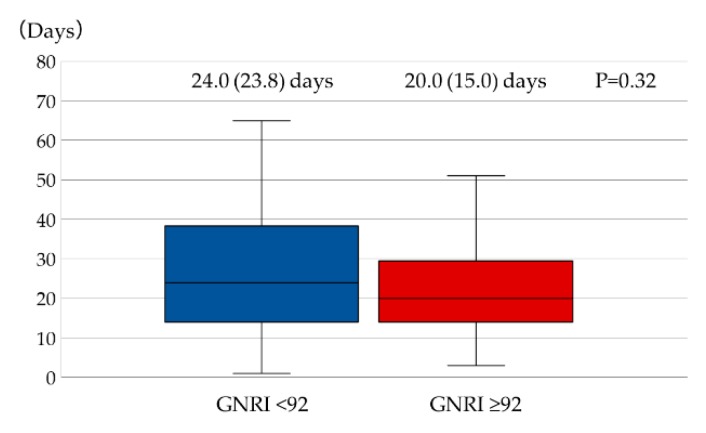
Comparison of length of hospital stay between patients with GNRI < 92 and ≥92 in HFrEF. There was no significant difference in the length of hospital stay between patients with GNRI < 92 and ≥92. Mann–Whitney U-test was used for comparison between the two groups. Abbreviations: GNRI, Geriatric Nutritional Risk Index; HFrEF, heart failure with reduced ejection fraction.

There was no significant difference (*p* = 0.32) in the length of hospital stay between patients with GNRI < 92 and ≥ 92 (median (IQR), 24.0 (23.8) days and 20.0 (15.0) days, respectively) (Figure 2). According to the univariate logistic regression analyses, GNRI < 92, NYHA class ≥ III, high potassium level, and low serum sodium, hemoglobin, and eGFR levels showed *p* values < 0.15. According to the multivariate analysis, NYHA class ≥ III and serum sodium level were determined as independent factors associated with longer length of hospital stay (i.e., >22 days) (Table 3).

### 3.3. Heart Failure with Preserved Ejection Fraction

#### 3.3.1. Baseline Characteristics

Characteristics of patients with HFpEF (*n* = 250) are shown in Table 4. Among them, 120 and 130 patients showed GNRI < 92 (48.0%) and GNRI ≥ 92 (52.0%), respectively. Patients with GNRI < 92 were significantly older and had lower body mass index, serum albumin level and GNRI than those with GNRI ≥ 92 (Table 4).

#### 3.3.2. In-Hospital Mortality

Of the 27 in-hospital mortalities, 16 (13.3%) were observed in patients with GNRI < 92 and 11 (8.5%) in patients with GNRI ≥ 92 (*p* = 0.23). According to the univariate logistic regression analyses, age, presence of diabetes, presence of atrial fibrillation, prior HF hospitalization, and low serum sodium, hemoglobin, and eGFR levels showed *p* values < 0.15. According to the multivariate analysis, prior HF hospitalization and eGFR levels were determined as independent factors associated with in-hospital mortality (Table 5).

#### 3.3.3. Length of Hospital Stay

**Figure 3:**
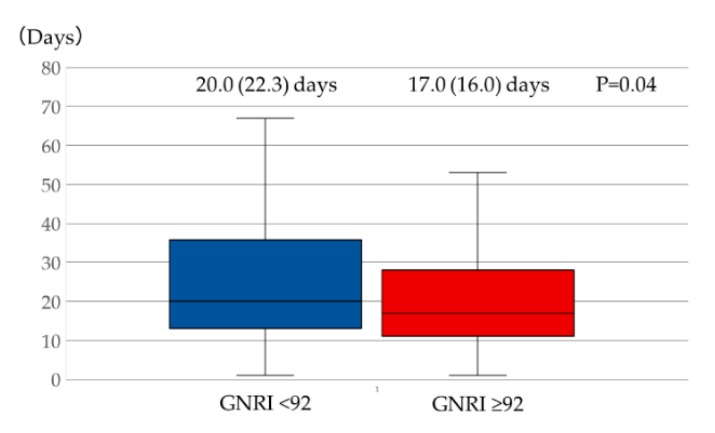
Comparison of length of hospital stay between patients with GNRI < 92 and ≥92 in HFpEF. Patients with GNRI < 92 showed longer length of hospital stay than those with GNRI ≥ 92. Mann–Whitney U-test was used for comparison between the two groups. Abbreviations: GNRI, Geriatric Nutritional Risk Index; HFpEF, heart failure with preserved ejection fraction.

Patients with GNRI < 92 had longer length of hospital stay than those with GNRI ≥ 92 (median (IQR), 20.0 (22.3) days and 17.0 (16.0) days, respectively; *p* = 0.04) (Figure 3). According to the univariate logistic regression analyses, GNRI < 92, male sex, presence of hypertension, ischemic etiology, use of diuretics, low serum potassium and hemoglobin levels, elevated CRP levels, and increased BNP levels showed *p*-values < 0.15. According to the multivariate analysis, GNRI < 92 and hemoglobin, BNP, and CRP levels were determined as independent factors associated with longer length of hospital stay (i.e., >19 days) (Table 6).

## 4. Discussion

In this study, several important findings were reported that provide insight into the association between nutritional status on admission and short-term outcomes in hospitalized patients with acute decompensated HF, specifically patients with HFpEF. First, in HFrEF, although serum sodium and hemoglobin levels were identified as independent factors associated with increased in-hospital mortality, there was no association between malnutrition expressed as GNRI < 92 and in-hospital mortality. Second, only serum sodium level was the independent factor associated with longer length of hospital stay, and no association was observed between malnutrition expressed as GNRI < 92 and length of hospital stay in patients with HFrEF. Third, in HFpEF, patients with history of prior HF hospitalization and patients with lower eGFR levels were likely to experience in-hospital mortality, but malnutrition expressed as GNRI < 92 was not a factor associated with increased in-hospital mortality. Finally, in HFpEF, GNRI < 92 was significantly associated with longer length of hospital stay and lower hemoglobin, higher BNP, and elevated CRP levels among patients hospitalized due to acute decompensated HF. Our findings suggest that nutritional status on admission assessed using the GNRI may be an important factor when stratifying patients at high risk for longer length of hospital stay in patients hospitalized due to acute decompensated HF, and this should be emphasized in patients with HFpEF.

Previous studies have reported that body mass index and serum albumin levels, which are traditional indicators of nutritional status, can predict mortality in patients with HF [17,18,19]. Additionally, more recently, some other indices that were basically computed by serum albumin level and lymphocyte count, such as the Prognostic Nutritional Index (PNI) and Controlling Nutritional Status (CONUT) score, have also been shown to have prognostic value in patients with HF, and more precisely assessed the prognostic values of malnutrition on long-term clinical outcomes in patients with HF [20,21]. These studies suggest that assessment of nutritional status is important in patients with stable HF. Additionally, a previous study has reported that in patients hospitalized due to acute decompensated HF, either a severe CONUT score or low PNI was associated with an increased risk of in-hospital mortality and prolonged length of hospital stay [22]. Thus, rapid assessment of nutritional status on admission is also important in patients hospitalized due to acute decompensated HF to stratify patients with high risks for in-hospital mortality and prolonged length of hospital stay. The GNRI, which comprises serum albumin level and ideal body weight, is a simpler index used to assess nutritional status than PNI or CONUT, considering that lymphocyte count is not assessed in the GNRI. It has been reported that the GNRI had the greatest incremental value in predicting the risk of poor prognosis in HF outpatients among the three scoring systems [23]. Although the clinical utility of the GNRI for predicting long-term mortality in patients with HF has already been reported in several studies [11,24,25], the association between GNRI and in-hospital mortality or length of hospital stay has rarely been investigated. In one study by Aziz and colleagues, nutritional status assessed by the Nutritional Risk Index, which also comprises serum albumin level and ideal body weight, was associated with prolonged length of hospital stay [26]. However, there are no studies investigating the impact of malnutrition expressed using GNRI on in-hospital mortality and length of hospital stay in patients hospitalized due to acute decompensated HF separately with HFrEF and HFpEF.

In a recent study by Nishino et al., serum albumin levels on admission were associated with prolonged length of hospital stay [27]. Thus, a novel observation of the present study is that in patients hospitalized due to acute decompensated HF, admission malnutrition based on the GNRI, which can more precisely assess nutritional status compared to other scoring systems, is associated with length of hospital stay in HFpEF but not in HFrEF. Although it is unclear why the GNRI failed to show a statistically significant relationship with either in-hospital mortality or length of hospital stay in patients with HFrEF, age difference may play an important role, as patients with HFrEF are younger than those with HFpEF. According to the Organization for Economic and Cooperation and Development report in 2017, the average length of hospital stay is markedly longer in Japan than in other countries. For patients with HF, a longer hospital stay is a serious problem considering that it negatively affects the patients’ quality of life and increases the patients’ healthcare costs [8]. Furthermore, HFpEF has recently become highly prevalent in patients hospitalized due to acute decompensated HF, possibly as a result of an increasing elderly population [28]. Thus, shortening the length of hospital stay for patients with acute decompensated HF and HFpEF is significantly required. Considering the fact that there are no established pharmacological treatments to improve clinical outcomes of HFpEF and that a more comprehensive approach might be necessary to improve the clinical outcomes of HFpEF [29], it may be worthwhile to investigate whether rapid nutritional intervention can shorten length of hospital stay in elderly patients with HFpEF and malnutrition.

The present study has several limitations. First, because it was an observational study, the results do not confirm the cause-and-effect relationship between malnutrition assessed by the GNRI and in-hospital mortality or length of hospital stay. Thus, further interventional studies are required to confirm this cause-and-effect relationship. Second, in patients with HF, both serum albumin level and body weight are influenced by non-nutritional factors such as fluid overload. Therefore, the measurement of albumin, body mass index, and body weight individually is unsuitable for nutritional assessment in patients with acute decompensated HF. Although using the GNRI as a nutritional indicator may overcome the individual limitations of each indicator [26], the association between the changes in each indicator during hospitalization and clinical outcomes should be assessed in a further study. Third, this study was a single-center, observational study and included a limited number of in-hospital mortalities. Finally, we cannot exclude the possibility that unmeasured factors may have influenced some of our findings, even after taking confounding factors into account. Thus, the findings of our study should be interpreted with caution.

## 5. Conclusions

Among patients hospitalized due to acute decompensated HF, an assessment of nutritional status on admission by GNRI was not a significant independent factor associated with increased in-hospital mortality, either in patients with HFpEF or HFrEF. Only in patients with HFpEF, GNRI <92, along with lower hemoglobin, higher BNP, and elevated CRP levels was the independent factors for longer length of hospital stay. GNRI is useful for stratifying patients at high risk for longer length of hospital stay in HFpEF but not in HFrEF. These observations provide important information, specifically when we consider an increasing elderly population [3] and its associated increasing prevalence of HF [2], particularly HFpEF.

## Figures and Tables

**Table 1:** Characteristics of patients with HFrEF.

	Total (*n* = 201)	GNRI < 92 (*n* = 96)	GNRI ≥ 92 (*n* = 105)	*p*
Age, years	67.1 ± 14.3	71.0 ± 12.0	63.6 ± 15.3	<0.01
Male sex	142 (70.7)	66 (68.8)	76 (72.4)	0.64
Body mass index, kg/m^2^	23.0 ± 5.2	20.5 ± 3.4	25.2 ± 5.6	<0.01
Current smoker	104 (51.7)	54 (56.2)	50 (47.6)	0.26
Hypertension	141 (70.2)	65 (67.7)	76 (72.4)	0.54
Diabetes mellitus	76 (37.8)	40 (41.7)	36 (34.3)	0.31
Ischemic etiology	88 (43.8)	46 (47.9)	42 (40.0)	0.32
Atrial fibrillation	65 (32.3)	30 (31.2)	35 (33.3)	0.77
Prior HF hospitalization	111 (55.2)	51 (53.1)	60 (57.1)	0.57
LVEF, %	27.1 ± 6.9	27.1 ± 7.1	27.0 ± 6.8	0.87
NYHA class ≥ III	175 (87.6)	85 (88.5)	90 (85.7)	0.68
Serum albumin, mg/dL	3.3 ± 0.6	2.9 ± 0.5	3.7 ± 0.4	<0.01
Serum sodium, mmol/L	138.6 ± 4.2	138.2 ± 4.3	138.9 ± 4.1	0.27
Serum potassium, mmol/L	4.2 ± 0.6	4.3 ± 0.8	4.2 ± 0.5	0.33
Hemoglobin, g/dL	13.0 ± 2.6	12.4 ± 2.6	13.5 ± 2.4	<0.01
eGFR, mL/min/1.73 m^2^	54.5 ± 26.2	52.0 ± 27.2	56.7 ± 25.1	0.20
BNP, pg/dL	1106.3 (770.0)	1269.4 (893.6)	950.0 (683.6)	<0.01
CRP, mg/dL	3.5 (0.9)	4.1 (1.0)	3.0 (0.9)	0.21
Elevated CRP	91 (45.3)	45 (46.9)	46 (43.8)	0.88
GNRI	93.1 ± 13.6	82.2 ± 6.7	103.1 ± 10.3	<0.01
**Medications**				
ACE-Is/ARBs	70 (34.8)	28 (29.2)	42 (40.0)	0.14
Aldosterone blockers	36 (17.9)	19 (19.8)	17 (16.2)	0.58
β-blockers	69 (34.3)	31 (32.3)	38 (36.2)	0.66
Diuretics	86 (42.8)	47 (49.0)	39 (37.1)	0.12

Variables are expressed as mean ± standard deviation, median (interquartile range), or number (%). ACE-I, angiotensin-converting enzyme inhibitor; ARB, angiotensin II receptor blocker; BNP, B-type natriuretic peptide; BP, blood pressure; CRP, C-reactive protein; eGFR, estimated glomerular filtration rate; GNRI, Geriatric Nutritional Risk Index; HF, heart failure; HFrEF, heart failure with reduced ejection fraction; LVEF, left ventricular ejection fraction; NYHA, New York Heart Association.

**Table 2:** Univariate and multivariate analyses of factors associated with increased in-hospital mortality in patients with HFrEF.

Variables	Univariate			Multivariate		
	OR	95% CI	*p*	OR	95% CI	*p*
GNRI < 92 (yes)	2.94	0.89–9.70	0.08	-	-	-
Diuretics (yes)	2.57	0.83–7.97	0.10	-	-	-
Serum sodium (1 mmol/L increase)	0.79	0.70–0.89	<0.01	0.81	0.71–0.91	<0.01
Hemoglobin (1 g/dL increase)	0.74	0.59–0.93	<0.01	0.78	0.61–1.00	0.04

CI, confidence interval; GNRI, Geriatric Nutritional Risk Index; HFrEF, heart failure with reduced ejection fraction; OR, odds ratio.

**Table 3:** Univariate and multivariate analyses of factors associated with length of hospital stay in HFrEF.

Variables	Univariate			Multivariate		
	OR	95% CI	*p*	OR	95% CI	*p*
GNRI < 92 (yes)	1.62	0.91–2.88	0.11	-	-	-
NYHA class ≥ III (yes)	5.32	1.74–16.2	<0.01	4.83	1.57–14.9	<0.01
Serum potassium (1 mmol/L increase)	1.76	1.03–2.99	0.04	-	-	-
Serum sodium (1 mmol/L increase)	0.89	0.82–0.97	<0.01	0.90	0.83–0.98	0.01
Hemoglobin (1 g/dL increase)	0.84	0.74–0.94	<0.01	-	-	-
eGFR (1 mL/min/1.73 m^2^ increase)	0.99	0.98–1.00	0.10	-	-	-

CI, confidence interval; eGFR, estimated glomerular filtration rate; GNRI, Geriatric Nutritional Risk Index; HRrEF, heart failure with reduced ejection fraction; NYHA, New York Heart Association; OR, odds ratio.

**Table 4:** Characteristics of patients with HFpEF.

	Total (*n* = 250)	GNRI < 92 (*n* = 120)	GNRI ≥ 92 (*n* = 130)	*p*
Age, years	71.5 ± 13.1	74.1 ± 12.6	69.0 ± 13.3	<0.01
Male sex	154 (61.6)	71 (59.2)	83 (63.8)	0.51
Body mass index, kg/m^2^	22.8 ± 4.4	20.2 ± 3.1	25.2 ± 4.2	<0.01
Current smoker	108 (43.2)	53 (44.2)	55 (42.3)	0.80
Hypertension	195 (78.0)	90 (75.0)	105 (80.8)	0.29
Diabetes mellitus	102 (40.8)	45 (37.5)	57 (43.8)	0.37
Ischemic etiology	92 (36.8)	41 (34.2)	51 (39.2)	0.43
Atrial fibrillation	96 (38.4)	45 (37.5)	51 (39.2)	0.80
Prior HF hospitalization	134 (53.6)	66 (55.0)	68 (52.3)	0.70
LVEF, %	56.0 (38.4)	55.6 (11.6)	56.3 (11.1)	0.67
NYHA class ≥ III	202 (80.8)	97 (80.8)	105 (80.8)	1.00
Serum creatinine, mg/dL	1.3 ± 1.0	1.4 ± 1.2	1.2 ± 0.8	0.11
Serum albumin, mg/dL	3.3 ± 0.6	2.9 ± 0.5	3.6 ± 0.5	<0.01
Serum potassium, mmol/L	4.2 ± 0.7	4.2 ± 0.7	4.2 ± 0.7	0.92
Serum sodium, mmol/L	138.8 ± 4.2	138.4 ± 4.3	139.1 ± 4.2	0.22
Hemoglobin, g/dL	11.9 ± 2.5	11.7 ± 2.4	12.1 ± 2.5	0.20
eGFR, mL/min/1.73 m^2^	53.7 ± 27.4	53.3 ± 30.9	54.1 ± 23.8	0.80
BNP, pg/dL	757.9 (517.6)	820.0 (553.6)	706.0 (462.5)	0.10
CRP, mg/dL	3.3 (0.9)	3.8 (0.9)	2.9 (0.7)	0.15
Elevated CRP	109 (43.6)	55 (45.8)	54 (41.5)	0.21
GNRI	92.3 ± 12.5	82.0 ± 7.1	101.8±8.2	<0.01
**Medications**				
ACE-Is/ARBs	103 (41.2)	45 (37.5)	58 (44.6)	0.30
Aldosterone blockers	28 (11.2)	13 (10.8)	15 (11.5)	1.00
β-blockers	72 (28.8)	30 (25.0)	42 (32.3)	0.21
Diuretics	93 (37.2)	46 (38.3)	47 (36.2)	0.79

Variables are expressed as mean ± standard deviation, median, or number (%). ACE-I, angiotensin-converting enzyme inhibitor; ARB, angiotensin II receptor blocker; BNP, B-type natriuretic peptide; BP, blood pressure; CRP, C-reactive protein; eGFR, estimated glomerular filtration rate; GNRI, Geriatric Nutritional Risk Index; HF, heart failure; HFpEF, heart failure with preserved ejection fraction; LVEF, left ventricular ejection fraction; NYHA, New York Heart Association.

**Table 5:** Univariate and multivariate analyses of factors associated with increased in-hospital mortality in HFpEF.

Variables	Univariate			Multivariate		
	OR	95% CI	*p*	OR	95% CI	*p*
Age (1-year increase)	1.04	1.00–1.08	0.03	-	-	-
Diabetes mellitus (yes)	2.32	1.03–5.23	0.04	-	-	-
Atrial fibrillation (yes)	0.42	0.16–1.09	0.07	-	-	-
Prior HF hospitalization (yes)	3.41	1.32–8.77	0.01	3.10	1.19–8.10	0.02
Serum sodium (1 mmol/L increase)	0.91	0.84–1.00	0.04	-	-	-
Hemoglobin (1 g/dL increase)	0.78	0.65–0.93	<0.01	-	-	-
eGFR (1 mL/min/1.73 m^2^ increase)	0.97	0.96–0.99	<0.01	0.97	0.96–0.99	<0.01

CI, confidence interval; eGFR, estimated glomerular filtration rate; GNRI, Geriatric Nutritional Risk Index; HF, heart failure; HFpEF, heart failure with preserved ejection fraction; OR, odds ratio.

**Table 6:** Univariate and multivariate analyses of factors associated with longer length of hospital stay in HFpEF.

Variables	Univariate			Multivariate		
	OR	95% CI	*p*	OR	95% CI	*p*
Male sex	0.66	0.38–1.14	0.14			
GNRI < 92 (yes)	1.71	1.01–2.92	<0.05	1.91	1.00–3.65	<0.05
Hypertension (yes)	0.50	0.27–0.95	0.04			
Ischemic etiology (yes)	0.55	0.31–0.97	0.04			
Diuretics (yes)	1.56	0.09–2.70	0.11			
Serum potassium (1 mmol/L increase)	0.72	0.48–1.08	0.12			
Hemoglobin (1 g/dL increase)	0.84	0.74–0.94	<0.01	0.83	0.72–0.95	<0.01
Log-transformed BNP (1 pg/dL increase)	2.57	1.36–4.85	<0.01	2.06	1.03–4.12	0.04
Elevated CRP (yes)	2.85	1.62–5.01	<0.01	3.03	1.59–5.78	<0.01

BNP, B-type natriuretic protein; CI, confidence interval; CRP, C-reactive protein; GNRI, Geriatric Nutritional Risk Index; HFpEF, heart failure with preserved ejection fraction; OR, odds ratio.
